# The Effect of Ethanol on Disassembly of Amyloid-β_1-42_ Pentamer Revealed by Atomic Force Microscopy and Gel Electrophoresis

**DOI:** 10.3390/ijms23020889

**Published:** 2022-01-14

**Authors:** Atsuya Matsui, Jean-Pierre Bellier, Takeshi Kanai, Hiroki Satooka, Akio Nakanishi, Tsukasa Terada, Takafumi Ishibe, Yoshiaki Nakamura, Hiroyasu Taguchi, Nobuyasu Naruse, Yutaka Mera

**Affiliations:** 1 Department of Fundamental Bioscience, Shiga University of Medical Science, Otsu 520-2192, Japan; ds111877@g.shiga-med.ac.jp (A.M.); ds111623@g.shiga-med.ac.jp (T.K.); hsatooka@belle.shiga-med.ac.jp (H.S.); nakanisi@belle.shiga-med.ac.jp (A.N.); mera@belle.shiga-med.ac.jp (Y.M.); 2 Molecular Neuroscience Research Center, Shiga University of Medical Science, Otsu 520-2192, Japan; taguti@belle.shiga-med.ac.jp; 3 Graduate School of Engineering Science, Osaka University, 1-3 Machikaneyama-Cho, Toyonaka 560-8531, Japan; u897541f@ecs.osaka-u.ac.jp (T.T.); ishibe.takafumi.es@osaka-u.ac.jp (T.I.); nakamura.yoshiaki.es@osaka-u.ac.jp (Y.N.)

**Keywords:** amyloid-β, oligomer, atomic force microscopy, gel electrophoresis, Gaussian fitting

## Abstract

The most common type of dementia, Alzheimer’s disease, is associated with senile plaques formed by the filamentous aggregation of hydrophobic amyloid-β (Aβ) in the brains of patients. Small oligomeric assemblies also occur and drugs and chemical compounds that can interact with such assemblies have attracted much attention. However, these compounds need to be solubilized in appropriate solvents, such as ethanol, which may also destabilize their protein structures. As the impact of ethanol on oligomeric Aβ assembly is unknown, we investigated the effect of various concentrations of ethanol (0 to 7.2 M) on Aβ pentameric assemblies (Aβp) by combining blue native-PAGE (BN-PAGE) and ambient air atomic force microscopy (AFM). This approach was proven to be very convenient and reliable for the quantitative analysis of Aβ assembly. The Gaussian analysis of the height histogram obtained from the AFM images was correlated with band intensity on BN-PAGE for the quantitative estimation of Aβp. Our observations indicated up to 1.4 M (8.3%) of added ethanol can be used as a solvent/vehicle without quantitatively affecting Aβ pentamer stability. Higher concentration induced significant destabilization of Aβp and eventually resulted in the complete disassembly of Aβp.

## 1. Introduction

The generation of the amyloid-β (Aβ) peptide is a critical event in the pathogenesis of Alzheimer’s disease (AD), the most common type of dementia [1,2,3]. The Aβ precursor encoded by the amyloid precursor protein gene is cleaved by γ-secretase complex into Aβ peptides of various sizes, among which those of the 40 (Aβ_1-40_) and 42 (Aβ_1-42_) amino acids are the most abundant [4,5]. Aβ_1-42_ is the principal species found in the brain lysates of patients with AD [6,7,8]. The presence of aggregated Aβ in the form of senile plaques is one of the histopathological hallmarks of AD [2,9], but there is an emerging consensus that the soluble Aβ_1-42_ oligomeric assemblies (dimers, trimers, and small oligomeric assembly but not monomers) are also involved in the development of AD [10,11,12].

In vitro oligomerization using synthetic Aβ_1-42_ peptides results in preparation consisting of various assemblies (oligomers and fibrils). This heterogeneity, probably due to transient meta-stability and different kinetic pathways [13,14,15,16], may jeopardize attempts to characterize Aβ_1-42_ oligomeric assemblies. Some Aβ_1-42_ oligomerization protocols have been shown to result in a preferential assembly generation [12,17]. For instance, Ahmed et al. have shown that when parameters such as temperature, pH, salt, and initial monomer concentration are carefully controlled, then the pentameric conformers are the predominant assembly formed. This could be used to establish a model system to evaluate the ability of chemical compounds to destabilize Aβ_1-42_ pentameric assemblies (Aβp); an essential step towards developing therapeutic strategies targeting the processes of Aβ_1-42_ assembly/disassembly, opening new avenues for the treatment of AD.

Most chemical compounds with the potential to interact on Aβ_1-42_ oligomeric assemblies are not soluble in water [18], therefore alcohols and other surface-active agents are needed as the solvent/vehicle. Several alcohols have been shown to effectively disassemble Aβ_1-42_ oligomeric assemblies by acting on the tertiary and quaternary structures, although the exact molecular mechanism remains unknown [13,19,20]. Other studies have reported the effect of another solvent, dimethyl sulfoxide (DMSO), on the conformation of Aβ_1-40_ and Aβ_1-42_ [21,22]. However, the effect of ethanol on already assembled Aβ_1-42_ oligomers has never been reported, even if ethanol has been shown to prevent Aβ aggregation and protect cultured cells against both Aβ-induced cell death and synapse damage [23,24].

In this context, the objective of this study was to determine the highest ethanol concentration that could be used as a solvent/vehicle without affecting Aβp stability.

Recent studies are showing that direct observation of real space with sub-nanometer resolution, such as scanning probe microscopy (SPM), is useful for accessing peptides, especially their structures [25,26]. Previously, we used SPM to study Aβ_1-42_ oligomeric assemblies and showed the diversity of oligomeric conformations in vitro [27]. Although SPM permits accurate visualization of Aβ_1-42_ oligomeric assemblies, reliable quantification is tedious. Here, we studied the effect of ethanol on Aβp conformation using a combination of gel electrophoresis and atomic force microscopy (AFM), one of the SPM, two affordable and available technologies in most routine laboratories. These methods were used to assess quickly and reliably a homogeneous population of Aβp (obtained under carefully controlled parameters), and then fulfilled the objective of this study.

## 2. Results

### 2.1. Electrophoresis

**Figure 1:**
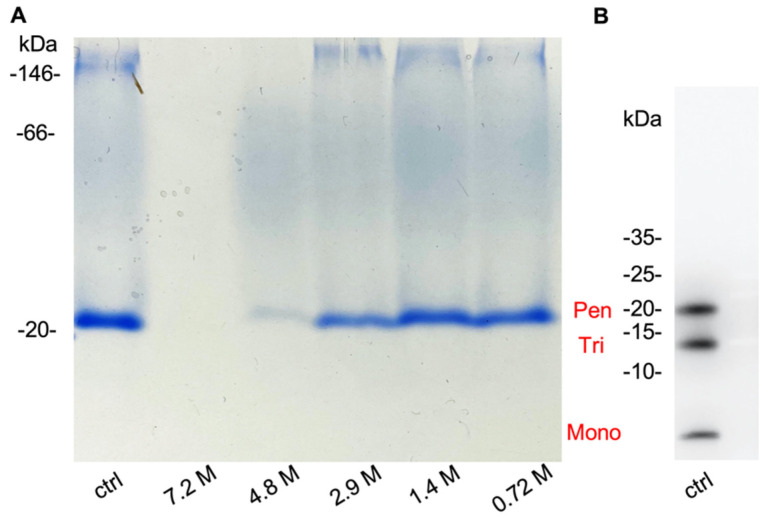
BN-PAGE and Tricine-SDS-PAGE of oligomerized Aβ_1-42_. (**A**) Blue-native PAGE of oligomerized Aβ_1-42_ incubated with various concentrations of ethanol for 3 h. “ctrl” is incubated with 0 M ethanol. (**B**) Tricine-SDS-PAGE of oligomerized Aβ_1-42_ solution in same conditions as “ctrl” in (**A**). Molecular weight markers are shown on the left of each gel image.

The reliability of Aβ_1-42_ oligomerization protocol was examined using polyacrylamide gel electrophoresis (PAGE) in both non-denaturing (native) and denaturing conditions. Aβ_1-42_ oligomeric assemblies were assessed in non-denaturing conditions using blue-native polyacrylamide gel electrophoresis (BN-PAGE) followed by Coomassie Brilliant Blue (CBB) staining (Figure 1A, ctrl). Aβ_1-42_ oligomeric assemblies were assessed in denaturing conditions using Tricine-SDS-PAGE, followed by Western blot analysis (Figure 1B, ctrl). In native gels, an intense sharp band with an apparent molecular size slightly above 20 kDa was observed; it was consistent with the expected size for Aβp. Faintly stained bands above 146 kDa and a smear between 20 and 146 kDa were also observed. On denaturing gels, oligomers are broken down by SDS, and three bands with apparent molecular sizes of 22.5 kDa, 14 kDa, and lower than 10 kDa were observed (Figure 1B). These bands matched the expected sizes for the Aβ_1-42_ pentamers, trimers, and monomers. Migration patterns on BN and Tricine-SDS gels were consistent with the results reported previously [17]. This confirmed that Aβp was the main product of oligomerization seen on BN-PAGE.

Next, we investigated the effect of various concentrations of ethanol on oligomerized Aβ_1-42_. After 3 h of incubation with ethanol (concentration from 0 to 7.2 M), the Aβ_1-42_ oligomerization status was examined by BN-PAGE (Figure 1A). The migration pattern was essentially similar to the control (without ethanol) for ethanol concentrations of up to 4.8 M; however, the intensity of the 20 kDa band obviously decreased as the ethanol concentration increased (Figure 1A). At the highest ethanol concentration tested (7.2 M), no stained band was visible. Later, densitometric analysis of bands (Figure 6A, white bars) confirmed that the 20 kDa band intensity significantly diminished when the ethanol concentration increased. These results suggest that ethanol definitely affected the stability of oligomerized Aβ_1-42_.

### 2.2. AFM Imaging and Height Distribution

**Figure 2:**
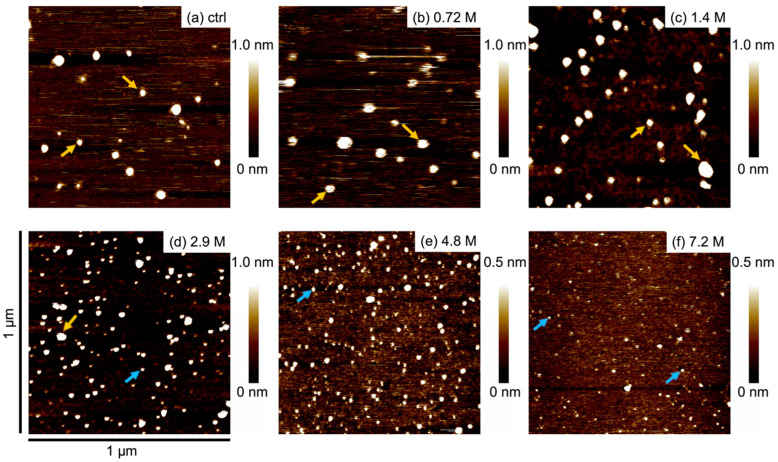
AFM images of oligomerized Aβ_1-42_ incubated with various ethanol concentration for 3 h: (**a**) control (0 M ethanol), (**b**) 0.72 M, (**c**) 1.4 M, (**d**) 2.9 M, (**e**) 4.8 M, and (**f**) 7.2 M. The height contrast is color-coded between 0.0–1.0 nm or 0.0–0.5 nm, as shown in the scale bar, with brighter colors indicating higher particle heights. The height scale was adjusted to a reference position. The yellow and blue arrows show pentamer and monomer, respectively. All AFM images are scaled at 1 × 1 squared micrometer; scale bars are shown in (**d**).

Imaging by AFM was used to resolve the spatial arrangement of oligomerized Aβ_1-42,_ in particular their heights. Figure 2a shows AFM resolved images of oligomerized Aβ_1-42_ after 3 h of incubation with various ethanol concentrations. In the control condition, AFM imaging of oligomerized Aβ_1-42_ showed about 10–20 relatively large-size (heights of 1.5–2.9 nm and widths of 1.8–4.6 nm) contrasted-spots (contrasts) per squared micrometers (Figure 2a). The number of large-size contrasts decreased while the number of small-size (heights of 0.5–1.2 nm and widths of 5.0–10 nm) contrasts increased after incubation with increasing ethanol concentrations (Figure 2b–f). These observations are consistent with the BN-PAGE results, showing that ethanol affected the stability of oligomerized Aβ_1-42_. It is noteworthy that AFM imaging clearly showed small contrasted spots after 3 h of incubation with the 7.2 M ethanol concentration, while BN-PAGE did not reveal any signal.

**Figure 3:**
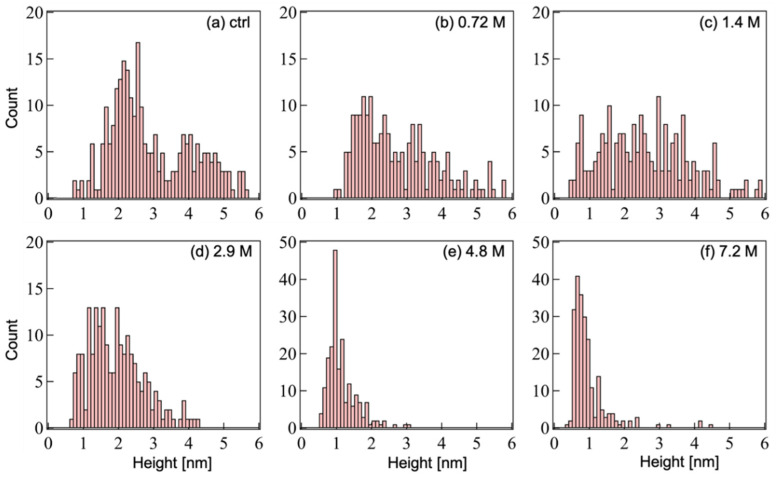
Height histograms of Aβ_1-42_ oligomeric assemblies calculated from the AFM topographic images in Figure 2. Ethanol concentrations: (**a**) control no ethanol, (**b**) 0.72 M, (**c**) 1.4 M, (**d**) 2.9 M, (**e**) 4.8 M, and (**f**) 7.2 M. The number of counted contrasts in each aliquot is more than 200.

Figure 3 shows the distribution of heights calculated from the AFM contrasts of oligomerized Aβ_1-42_ after incubation with ethanol. For ethanol concentrations ranging from 0 to 1.4 M, the heights of resolved contrasts are distributed up to 6 nm (Figure 3a–c). When the ethanol concentration exceeded 2.9 M (Figure 3d), most contrasts failed under 4 nm in height, and for concentrations over 4.8 M (Figure 3e,f), the resolved contrasts were typically below 2 nm in height with a dense distribution in the range of 0.4–1.8 nm.

### 2.3. Distribution Height Analysis by Gaussian Fitting

**Figure 4:**
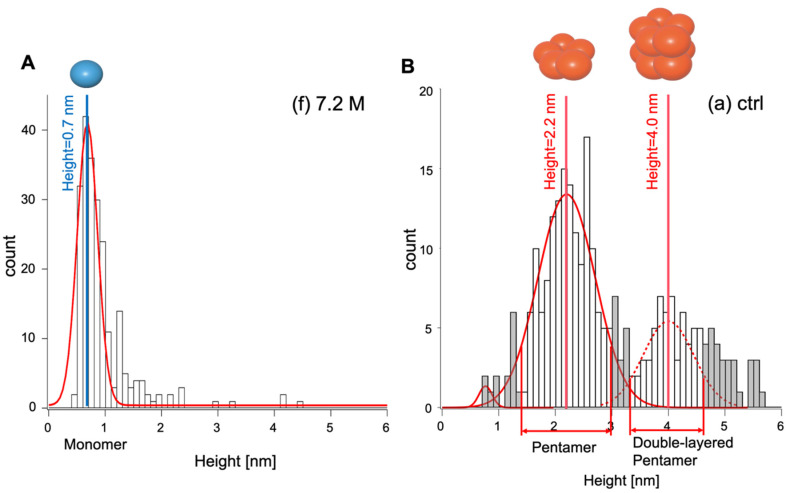
(**A**) Gaussian fitting for the histogram in Figure 3f. Values of mean and standard deviation are used for the calibration of the monomer. (**B**) Gaussian fitting applied to the histogram of Figure 3a. Bars in white are included in the pentamer counts.

In order to quantify distinct types of Aβ_1-42_ oligomeric assemblies based on their height features, we first characterized the signature feature of the monomeric and pentameric assemblies in the height histogram plot of Figure 3. Therefore, we used Gaussian fitting to resolve overlapping heights of peaks, and then we attempted the identification of Aβ_1-42_ oligomeric assemblies associated with resolved peaks, as described by Lin et al. [28]. First, Gaussian fitting was applied to the histogram corresponding to the smallest contrasted spots (Figure 3f); this was obtained at the highest ethanol concentration. The resulting Gaussian fitted curve peaked at a value of 0.69 ± 0.26 nm (mean ± standard deviation; Figure 4A). This corresponded to contrasts with heights of 0.5–1.2 nm and widths of 5.0–10 nm that we categorized as monomers. Next, the same procedure was applied to the histogram of Figure 3a corresponding to the largest and highest contrasted spots observed in control condition (without ethanol), which we assumed to be pentamers in regards to BN-PAGE data. Gaussian fitting resolved two curves peaking at 2.2 ± 0.71 nm and 4.0 ± 0.61 nm (Figure 4B). As suggested by Ahmed et al. [17], we assumed that there were pentamer and double-layered pentamers, respectively. It is important to note that the monomeric height values observed in this study were consistent with those previously reported for the Aβ_1-40_ monomer [12,29]. In addition, single layer and double layer Aβp height values closely matched those reported in previous studies [17,30,31,32].

**Figure 5:**
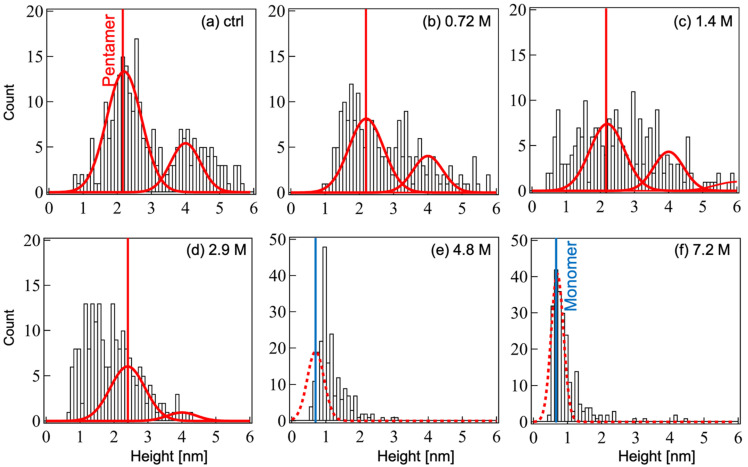
Characterization of Aβ_1-42_ oligomeric assembly by Gaussian fitting and calibration. Ethanol concentration: (**a**) control (no ethanol), (**b**) 0.72 M, (**c**) 1.4 M, (**d**) 2.9 M, (**e**) 4.8 M, and (**f**) 7.2 M. The solid red curves are Gaussian curves with peaks at 2.2 nm and 4.0 nm, and the broken red curves are those at 0.7 nm and 1.4 nm. Blue and red vertical lines are drawn at the values of 0.7 nm and 2.2 nm, respectively.

After Gaussian fitting, this calibration was applied to characterize the monomers, pentamers, and double-layered pentamers peaks histograms; this resulted in Figure 5. Similar to BN-PAGE, Aβp forms (single- and double-layered) decreased and monomeric Aβ_1-42_ increased when the ethanol concentration increased.

### 2.4. Combined AFM and Electrophoresis

**Figure 6:**
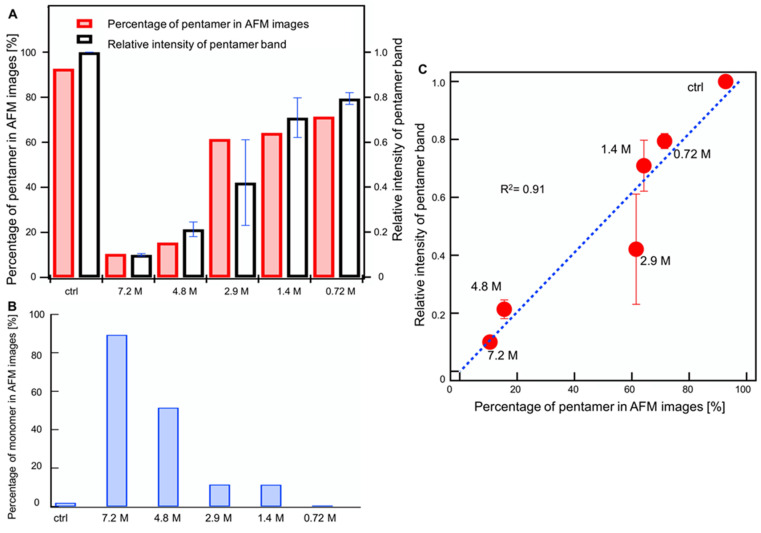
(**A**) Comparison of the pentameric percentage from AFM images (red bar) with band intensities in BN-PAGE (white bar). The error bar of bands represents the mean ± standard error of the mean (*n* = 3). (**B**) Count of monomers in AFM images. (**C**) Pearson correlation coefficient between the pentameric percentage from AFM images and the average ratio of pentameric band intensity in BN-PAGE.

The percentage of pentamers from the AFM resolved height was compared to the 20 kDa band intensity observed by BN-PAGE and was measured by software ImageJ (Figure 6A). Pearson’s correlation coefficient between the ratio of the number of pentamers in the height histograms and intensities of pentameric bands obtained from three independent BN-PAGE gels was 0.91, indicating a strong correlation between the BN-PAGE and AFM results (Figure 6C).

Figure 6B shows the percentage of monomers counted from the histogram height comprised between 0.69 nm ± 0.26 nm for all concentrations of ethanol tested. The percentage of monomers was inversely proportional to the percentage of pentamers (Figure 6A, red bars), indicating that as the concentration of ethanol increased, pentamers decreased and monomers increased. This result indicates that ethanol promoted the disassembly of pentamers into monomers.

## 3. Discussion

In the present study, we assessed the effect of ethanol on Aβp. We used a protocol of Aβ oligomerization, which results in predominant pentameric assembly generation when parameters such as temperature, pH, salt, and initial monomers concentration are carefully controlled [15,33]. Aβp generation was confirmed in the present and in previous studies [17,27]. The potential mechanisms liable for the preferential generation of Aβp were discussed elsewhere [13,34]. Next, to answer our initial objective to determine which concentration of ethanol can be used as solvent/vehicle without destabilizing Aβp assemblies, we investigated the effect of various concentrations of ethanol ranging from 0 to 7.2 M (0~41.4%) on Aβp assemblies stability using BN-PAGE and ambient air AFM. Our observations indicated that ethanol concentrations as high as 2.9 M (16.6%) induced significant changes in Aβp stability, promoted disassembly, and eventually resulted in the complete disassembly of Aβp into monomers at the higher concentration used (i.e., 7.2 M (41.4%)). This is relevant with the fact that Aβp assemblies are mostly built on hydrophobic inter-and intramolecular non-covalent interactions [10,35,36], and amphiphilic small molecules like ethanol might affect the stability of Aβp by acting as surface-active agents [37,38,39]. Interestingly, ethanol concentrations up to 1.4 M (8.3%) did not have much effect and could be used as a solvent/vehicle for drugs and chemical compounds to study their effects on Aβp stability. In a previous study, Ormeno et al. [23] reported that an ethanol concentration as low as 10 mM was able to prevent the build-up of Aβ_1-40_ aggregates, an almost 100 times lower ethanol concentration than what was required here to disassemble Aβp. This indicates that interfering with Aβ oligomerization required a lower ethanol concentration than that needed to disassemble the already build up Aβ oligomeric assembly. Finally, a study using DMSO as a solvent for Aβ_1-42_ has shown that DMSO up to 10% does not affect Aβ_1-42_ oligomeric assembly stability; however, it has also been reported that DMSO may oxidate Aβ_1-42_ methionine [40].

The above finding answered our initial question, however, several additional observations deserved further discussion. We used BN-PAGE and ambient air AFM to investigate the effect of ethanol on Aβp assembly—these are two affordable technologies that are relatively easy to set up in a routine biochemical laboratory. Interpretations of individual BN-PAGE and AFM datasets showed limitations, but their combination permitted a reliable quantitative assessment of Aβp stability. BN-PAGE followed by Coomassie blue staining has been previously used to quantify Aβp pentamers [17,27]. On our hand, we observed that BN-PAGE was unable to reveal low molecular weight Aβ assembly (i.e., monomers, dimer, trimer, and tetramer). The reason for this shortcoming is unclear; it could be due to the lack of sensitivity of Coomassie blue staining, the lack of resolution of the gel system used, or both [41]. On the other hand, AFM was very effective for a quick visual assessment. It could also identify Aβ oligomeric forms after cross-sectional height analysis by Gaussian fitting, and calibration using monomeric Aβ and Aβp profiles. With this original approach, we were able to follow Aβp disassembly when the ethanol concentration increased. The correlation between band intensity by BN-PAGE and pentamers count by AFM demonstrated that in our experimental setting, BN-PAGE could be used to quickly and reliably quantify Aβp. The combination of AFM and BN-PAGE may be an alternative approach to the actual methodologies for relating the molecular weight of peptides to their size and spatial organization, being able to quickly estimate their relative amounts [42].

The histogram of the height distribution of Aβp in the absence of ethanol showed two peaks. Based on previous observations, we interpreted them as single-layered Aβp (Figure 4B left peak red line) and double-layered Aβp (Figure 4B right peak red line) [17,27,31]. In contrast, the BN-PAGE analysis showed only a single band with a molecular size corresponding to that of a single-layered pentamer. This difference suggests either that double-layered Aβp is an artifact generated during processing the sample for AFM or that double-layered Aβp breaks down into single-layered Aβp during BN-PAGE migration. It was previously reported that during sample preparation for AFM observation, Aβ_1-42_ dispersed on mica could form oligomers and fibrils due to the presence of residual water [30,43,44]. In our experimental setting, when Aβ monomers were incubated for a short time, then only single-layered Aβp were observed using AFM (Supplementary Figure S1). This indicates that double-layered Aβp was not an artifact in our preparation. Alternatively, dyes have been shown to break down amyloid fibrils, suggesting that CBB (present in the loading buffer of BN-PAGE) may break down double-layered Aβp into single-layered Aβp [45]. Noteworthily, we observed that the actual best correlation between band intensity after BN-PAGE and pentamers count after AFM were obtained when pentamers count included the number of single-layered Aβp plus twice the number double-layered Aβp. Attempts to correlate BN-PAGE band intensity with the count number of single-layered Aβp only resulted in a weaker correlation (Supplementary Figure S2). This assumption suggests that the interactions keeping Aβp stacked together to form double-layered Aβp are weaker than the interactions making the Aβp assembly. This supports the computer simulation predicting that Aβp models can be stacked to form a loosely packed decameric Aβ (double-layered Aβp) through a small number of hydrophobic interactions [31].

In line with the previous assumption, we described a drastic decrease in the number of double-layered pentamers in the presence of ethanol concentrations as high as 2.9 M (16.6%), then complete disassembly of Aβp into monomeric Aβ at the highest ethanol concentration used (i.e., 7.2 M (41.4%)). It was tempting to reconstitute the sequence of double-layered Aβp break down and Aβp disassembly, however, it appears very complex. Many intermediate heights between double-layered Aβp and single-layered heights suggested that a molecular intermediate existed, maybe mixed double-layered with Aβp and another Aβ form (monomer, dimer, trimer, and tetramers). While we were able to calibrate the Aβp and Aβ monomer height peaks, other heights were difficult to reliably identify; as a result, the sequence of events leading to Aβp disassembly was not resolved and will require further studies.

In conclusion, we found that ethanol concentrations up to 1.4 M (8.3%) have no significant effect on single-layered Aβp stability and can be used as a solvent/vehicle for drugs and chemical compounds. We showed that a combination of AFM and BN-PAGE, two technologies readily available and affordable, might provide new insight facilitating both qualitative and quantitative assessment of the Aβ oligomeric forms. Interestingly, we showed that ethanol concentrations as high as 2.9 M (16.6%) were able to disassemble already formed Aβp assemblies.

## 4. Materials and Methods

### 4.1. Materials

Amyloid-β_1-42_ (human, Trifluoroacetate) was purchased from Peptide Institute (Osaka, Japan); UV/mass spectrometry high-performance liquid chromatography and amino acid analysis provided by the maker indicated 95% purity and confirmed the identity of the peptides. Low-binding microtubes and tips were used for all of the procedures involving peptides. All of the reagents were purchased from Nacalai Tesque (Kyoto, Japan) unless otherwise indicated. Ethanol (99.5%) was converted into moles from the indicated values according to the International Alcohol Table based on the International Organization of Legal Metrology (https://www.oiml.org/en/files/pdf_r/r022-e75.pdf) (accessed on 12 December 2021).

### 4.2. Aβ Oligomer Sample Preparation

Amyloid-β oligomers were generated according to the procedure described by Ahmed et al. [17]. First, Aβ_1-42_ monomers were prepared by dissolving peptides, with gentle pipetting, in hexafluoroisopropanol (HFIP, Fujifilm, Osaka, Japan) to a final concentration of 3.3 mg/ml. The resulting solution was transferred into a microtube, and the solvent was removed by vacuum freeze-drying. Lyophilized Aβ_1-42_ monomer preparation was dissolved in 1020 µL of a buffer solution consisting of 3.5% of 0.1 M NaOH (aqueous solution) in a 0.05 M phosphate buffer (pH 7.4) containing 0.05 M NaCl (Fujifilm, Osaka, Japan). The resulting 0.12 mM Aβ_1-42_ solution was aliquoted in six equal parts into microtubes and was incubated for 3 h at 4 °C. Next, each aliquot was incubated for another 3 h at 4 °C with various ethanol concentrations (Table 1). The Aβ_1-42_ concentration was adjusted to 72 mM in each tube by adding a cold buffer solution for a final volume of 290 µL. For AFM, the samples were used immediately; for BN-PAGE and Tricine-SDS-PAGE, all of the aliquots were quickly frozen in liquid nitrogen and stored at −80 °C until use.

### 4.3. Electrophoresis

Blue-native PAGE was carried out as described by Schägger and von Jagow [46], with slight modifications. We introduced two modifications to the original BN-PAGE protocol [45]—the use of 10–20% gradient gel and the addition of Coomassie blue into the loading buffer. It improved the sharpness of the bands, resulting in more accurate quantification and molecular weight determination. Gradient mini-gels (9 × 7 cm) were prepared using acrylamide/bis-acrylamide mixed solution (37.5:1; Fujifilm, Osaka, Japan) in 300 mM Bis-tris buffer (pH 7.0) containing 400 mM 6-aminocaproic acid. The stacking layer consisted of 4% acrylamide/bis-acrylamide mixed solution in the same buffer as above. Immediately after thawing, 50 μL of AβOs solutions were added to 25 μL Native PAGE sample buffer (Thermo Fisher Scientific, Ann Arbor MI, USA) containing 0.05% CBB G250. Then, 10 μL was loaded onto the 10–20% gradient gel and it was electrophoresed for 60 min at 70 V followed by 50 min at 200 V; cathode and anode running buffers were used (NativePAGE™ Cathode Buffer Additive and NativePAGE™ Running Buffer, Thermo Fisher Scientific, Ann Arbor, MI, USA). Native-Mark unstained protein standards (Thermo Fisher Scientific, Ann Arbor, MI, USA) were used as the molecular weight markers. After electrophoresis, gels were stained with Coomassie blue dye (rapid stain CBB kit, Nacalai-Tesque, Kyoto, Japan) for 1–3 days and were washed extensively with Milli-Q water to remove the background dark blue color. The gels were set on a LED lightbox (Konica, Tokyo Japan), and the images were acquired using an iPhone (Apple, Cupertino, CA, USA). The images were adjusted for contrast and luminosity only, and the band intensity was quantified using ImageJ [47,48] and calculated as relative values compared with the control lane (no ethanol).

Tricine-SDS-PAGE was performed following the method described by Schägger [49]. Immediately after thawing, 10 µL AβOs solutions were mixed with 50 µL non-reducing sample buffer solution (Fujifilm, Osaka, Japan) and loaded onto Novex 10–20% Tricine gels (Thermo Fisher Scientific, Ann Arbor, MI, USA). Nacalai Ladder-one plus stained protein standards were used as the molecular weight markers. Gel electrophoresis was carried out for 15 min at 60 V, for 60 min at 90 V, and for 60 min at 80 V using running cathode (0.1 M Tris-tricine and 1% SDS, pH 8.25) and anode (0.1 M Tris, pH 8.9) buffers. Separated peptides were transferred onto a PVDF membrane for Western blot analysis, as previously described [50]. The membrane was blocked in 5% skimmed milk dissolved in Tris-buffered saline with Tween 20 (TBST; 20 mM Tris-HCl, pH 7.6, 150 mM NaCl, 0.1% Tween 20) for 30 min. The blocked membrane was incubated overnight at room temperature with a 100 ng/ml anti-Aβ_1-16_ antibody in TBST containing 1% skimmed milk. Anti-Aβ_1-16_ is a purified mouse monoclonal IgG1, κ antibody (Biolegend, San diego, CA, USA; clone 6E10, CAT#803001, LOT#B247600), which has been validated for Western blot application. After incubation, the membrane was washed 3 × 10 min in TBST, then incubated for 1 h at room temperature with secondary peroxidase-labeled rabbit anti-mouse IgG(H+L) antibody (Jackson ImmunoResearch Laboratories, West Grove, PA, USA) diluted 1:20,000 in TBST. After further washes in TBST, the membrane was incubated for 1 min with a Chemi-Lumi One Super chemiluminescent substrate. Images were acquired using FUSION solo-S system (Vilber-Lourmat, Collégien, France), with settings to ensure an optimal dynamic range. The images in the figures were adjusted for contrast and luminosity only.

### 4.4. AFM Observations

Immediately after incubation with ethanol, aliquots of AβOs solution were diluted 200-times with Milli-Q, and 8 μL were dispersed onto a freshly cleaved mica disc (V-1 Grade 9.5 nm φ × 0.15 nm, Alliance Biosystems Co., Ltd., Osaka, Japan), which we confirmed as being ideally flat (height < ±0.3 nm). This procedure was performed to reduce the risk that the buffer solution changes the hydration landscape of Aβ_1-42_ and that potassium carbonate crystallites can be present on mica surface [51,52]. After drying in an antistatic case at 4 °C in a dry environment, observations were carried out using an AFM Innova system (Bruker, Billerica, MA, USA), operating in the air under ambient humidity and room temperature conditions. Mica discs were examined with AFM using the tapping mode. The resonant frequency was about 110 kHz. Areas of 1 × 1 micrometer and 512 × 512 pixels were acquired using software NanoDrive (v8.02, Bruker, Billerica, MA, USA) by scanning at 0.7 Hz. A silicon cantilever (RTESP-150, Bruker, Billerica, MA, USA) with a spring constant of 5 Nm/s (nominal value) and a tip radius of ~12 nm was used. Minimal resolutions in the x-, y-, and *z*-axis (height) were 0.1 nm and 0.1 nm, respectively.

### 4.5. Image and Histogram Analysis

Images obtained by AFM were analyzed with Gwyddion software (http://gwyddion.net/ version 2.56 (accessed on 12 January 2022)) [53]. To derive the true height of Aβ_1-42_ oligomeric assemblies by eliminating the effects of surface corrugation and scan noises, a cross-sectional analysis was conducted to obtain the actual height of the Aβ_1-42_ oligomeric assemblies in the AFM images fitted by Gaussian using Igor Pro 8 (HULINKS Inc., Tokyo, Japan) [54]. Several AFM images were acquired to generate the height histograms [25,55,56].

Gaussian fitting analyses for height histograms were based on the work of Lin, Y.C., and were also calculated using Igor Pro 8 (HULINKS Inc., Tokyo, Japan) [28]. To characterize the heights of the Aβ_1-42_ oligomeric assemblies, normalized height distributions were fitted by two or more Gaussian functions (Supplementary Figure S3).

For the correlation study, the total number of pentamers interpreted from the histograms was counted based on the pentamer height size of 1.5–2.9 nm and 3.4–4.6 nm for single and double-layered pentamers, respectively. The total population of pentamers was counted as the number contrast in the height range of 1.5–2.9 nm plus twice the number in the height range of 3.4–4.6 nm (double-layered pentamers).

## Figures and Tables

**Table 1:** Molar amounts of Aβ_1-42_ and ethanol in the samples. The ratio of Aβ_1-42_/ethanol id defined as the molar ratio. Aβ_1-42_ solution is considered uniform by sufficient stirring and dispersion, and the molar amount is calculated assuming that after HFIP treatment, the content of vials is exclusively monomers.

Aβ_1-42_ Peptide [nmol]	21	21	21	21	21	21
Ethanol [µL]	0 (ctrl)	120	80	48	24	12
Ethanol [M]	0	7.2	4.8	2.9	1.4	0.72
Ethanol [%]	0	41.4	27.6	16.6	8.3	4.1
Molar Ratio (Aβ_1-42_/ethanol)	-	1.0 × 10^−5^	1.5 × 10^−5^	2.5 × 10^−5^	5.0 × 10^−5^	1.0 × 10^−4^

## Data Availability

The data presented in this study are available upon request from the corresponding authors.

## Supplementary Materials

The following supporting information can be downloaded at: https://www.mdpi.com/article/10.3390/ijms23020889/s1.
