# Supplementary material for: The Effect of Ethanol on Disassembly of Amyloid-β1-42 Pentamer Revealed by Atomic Force Microscopy and Gel Electrophoresis
Source: Int J Mol Sci. 2022 Jan 14;23(2):889. doi: 10.3390/ijms23020889 (PMC8779648; doi:10.3390/ijms23020889)
Supplement: Supplementary file 1 [file ijms-23-00889-s001.zip › ijms-1529050-supplementary.pdf]

Supplementary Information

Supplementary Figure S1A, B

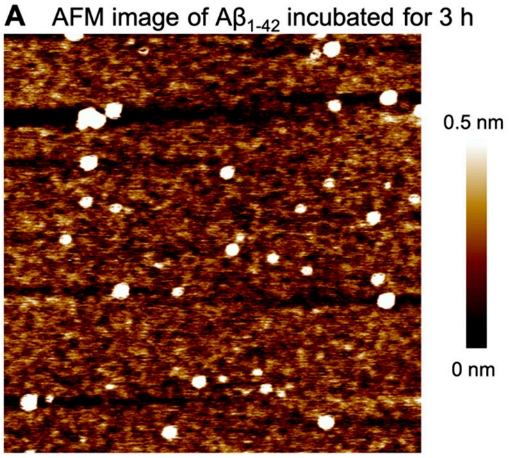

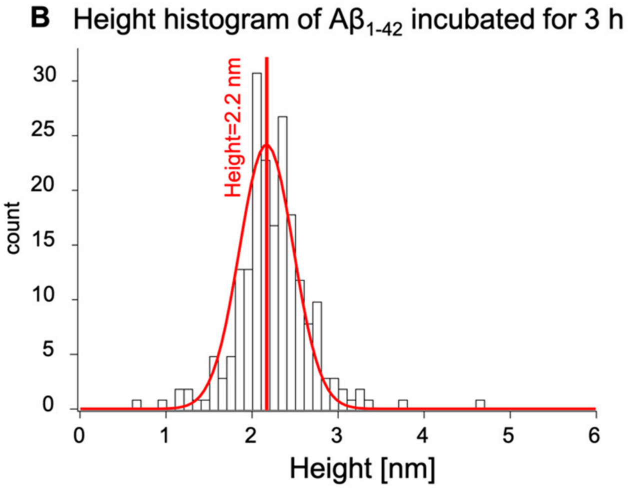

A typical AFM image (A) and height histogram (B) of $A\beta_{1-42}$ incubated for 3 hours. The value of height average is 2.2 ± 0.4 nm (mean ± standard deviation). The histogram B indicates mostly single-layered Aβp. When compared with AFM image and histogram of Aβ1-42 incubated for 6 hours (Figure 2 (a) ctrl, Figure 5 (a) ctrl) there is almost no double-layered Aβp, indicating that double-layered pentamers appeared after 3 hours incubation.

Supplementary Figure S2

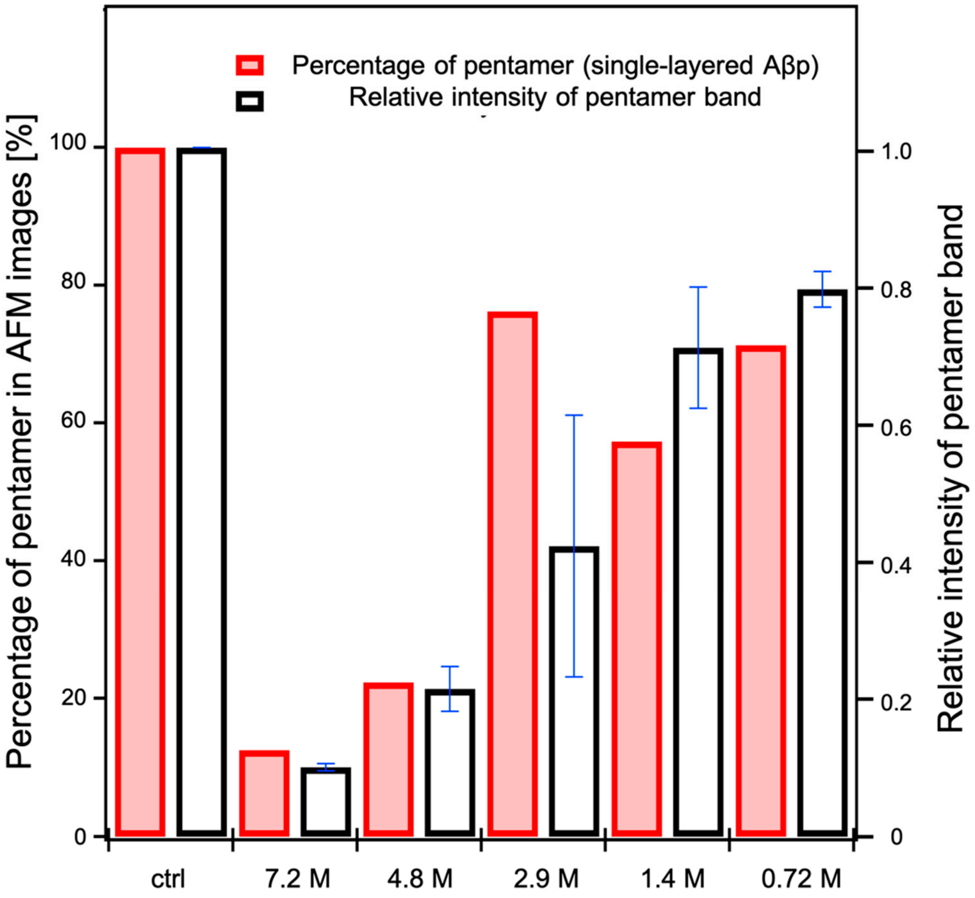

Comparison of pentamer ratio from AFM images (red bar) as in Figure 6A, when only single-layered Aβp were counted. The correlation with BN-PAGE band intensity (white bar) was weaker than when both single- and double-layered were included in the count (Figure 6A). Moreover, the heights of the single-layered and double-layered Aβp obtained in Figure 4B fit well with the past studies [17, 30-32, in main text reference].

Supplementary Figure S3

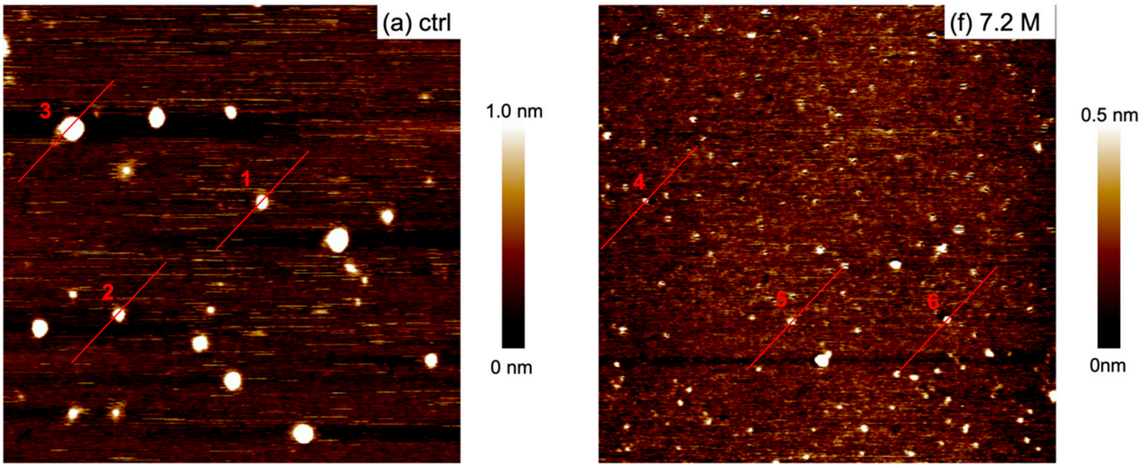

	1	2	3
height [nm]	2.1	2.2	4.7
width [nm]	10.8	13.0	19.3

	4	5	6
height [nm]	0.66	0.7	1.4
width [nm]	7.6	5.1	5.8

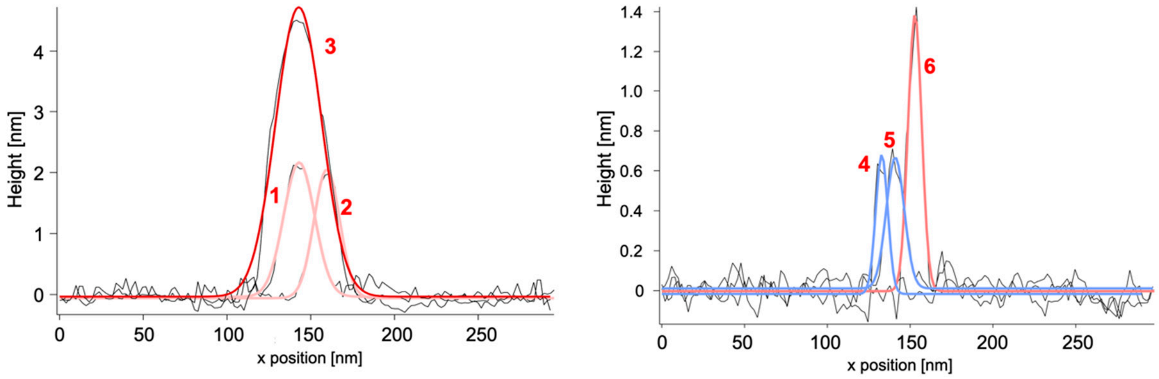

The Cross-sectional analysis was performed by drawing a cross line for each circular contrast as shown in the above figures (applied to Figure 2 (a) and (f) as an example in Supplementary Figure 3.). More than 200 line-profiles were drawn manually for each aliquot. To eliminate the effects of mica surface roughness and contaminants, Gaussian curve fitting was applied to all cross lines. The height values with multi-peak fitting by Gaussian curve were obtained using Igor Pro 8. This process was applied to all AFM images.
